# Hypocomplementemia in primary Sjogren’s syndrome: association with serological, clinical features, and outcome

**DOI:** 10.1007/s10067-022-06135-w

**Published:** 2022-03-29

**Authors:** Wei Lin, Zhifei Xin, Jialan Wang, Xiuying Ren, Yixuan Liu, Liu Yang, Shaoying Guo, Yupeng Yang, Yang Li, Jingjing Cao, Xiaoran Ning, Meilu Liu, Yashuang Su, Lijun Sun, Fengxiao Zhang, Wen Zhang

**Affiliations:** 1 Department of Rheumatology and Immunology, Hebei General Hospital, No. 348 Heping West Road, Shijiazhuang, 050051 Hebei China; 2 Department of Thoracic Surgery, Hebei General Hospital, Shijiazhuang, 050051 China; 3 Department of Gastroenterology and Nephrology, People’s Hospital of Huili County, Liangshan Yi Autonomous Prefecture, 615100 Sichuan Province China; 4 Department of Graduate School, Hebei Medical University, Shijiazhuang, 050017 China; 5 Department of Stomatology, Hebei General Hospital, Shijiazhuang, 050051 China; 6 Department of Oncology, Hebei General Hospital, Shijiazhuang, 050051 China; 7 Department of Rheumatology, Peking Union Medical College Hospital, Chinese Academy of Medical Science & Peking Union Medical College, National Clinical Research Center for Dermatologic and Immunologic Diseases, State Key Laboratory of Complex Severe and Rare Diseases, Beijing, 100730 China

**Keywords:** Hypocomplementemia, NK cell, Primary Sjögren syndrome, Risk factor, Systemic lupus erythematosus

## Abstract

**Objective:**

The aim of the present study was to assess the clinical characteristic of hypocomplementemia (HC) in primary Sjogren’s syndrome (pSS), and to address possible risk factors and the prognosis associated with HC in pSS patients.

**Methods:**

pSS patients with HC in Hebei General Hospital from September 2016 to March 2019 were retrospectively analyzed and compared to those with normocomplementemia (NC). Logistic regression analysis was used to detect risk factors.

**Results:**

Of the 333 patients with pSS, 84 patients (25.23%) were presented with HC at diagnosis. The presence of hyper-IgG and anti-Ro52 antibodies was significantly more common in patients with HC. In addition to systemic involvement, pSS patients with HC had more hematological, renal, and nervous system involvement, and received more immunosuppressant treatments than NC group (*p* < 0.05). ESSDAI score was significantly higher in patients with HC (*p* < 0.05). Multivariate logistic analysis indicated that leukopenia (OR = 2.23) and hyper-IgG (OR = 2.13) were independent risk factors for pSS with HC. In addition, profound CD16/CD56+ NK-cell lymphopenia was found in pSS-HC patients. More pSS patients developed SLE in the HC group than NC group (4.76% vs. 0.80%, *p* = 0.04) during the follow-up.

**Conclusion:**

HC was not an uncommon manifestation of pSS and had an independent association with the main clinical and immunological features. Patients with pSS-HC had an increased possibility to develop SLE that required more positive treatment with glucocorticoids and immunosuppressants.

**Key Points:**

• *Hypocomplementemia had an independent association with the main clinical and immunological features in primary Sjogren’s syndrome patients.*

• *ESSDAI score was significantly higher in patients with hypocomplementemia.*

• * The pSS patients with hypocomplementemia had an increased possibility to develop SLE.*

**Supplementary Information:**

The online version contains supplementary material available at 10.1007/s10067-022-06135-w.

## Introduction

Primary Sjögren’s syndrome (pSS) is a systemic autoimmune rheumatic disease characterized by lymphocytic infiltration of exocrine glands, which could lead to heterogeneous and various clinical presentations from sicca symptoms to systemic disease [1]. Approximately one-third of patients with pSS present extra-glandular manifestations, and some of these symptoms (e.g., interstitial lung disease, central nervous system [CNS] disease, and renal tubular acidosis) are associated with increased mortality. Abnormal immune response of T and B cells [2–4] recognition of self-antigens (Ro/SSA, La/SSB, and others), and subsequent activation are crucial for the cascade of events leading to the pSS pathology development [5, 6].

To date, there is increased evidence on distinct clinical phenotypes corresponding to unique autoantibodies. Various antibodies (e.g., anti-SSA, anti-SSB, anti-centromere antibodies [ACA]) have been reported to be classically correlated with parotid enlargement, Raynaud’s disease, arthritis, vasculitis, renal tubular acidosis, peripheral neuropathy, and cytopenias [7–11]. Similarly, several serologic abnormalities, such as B-cell proliferation, present special clinical features; for instance, hypergammaglobulinemia is often accompanied by extraglandular manifestations, especially with cutaneous vasculitis and pulmonary, articular, and renal involvements [12–15]. To date, few studies have addressed the clinical manifestations of hypocomplementemia (HC) or prognostic factors. This condition is not frequently observed but reported as a prognostic marker for lymphoma development in patients with pSS [16, 17].

Here, we described the clinical features and outcomes of patients with pSS with HC as the presenting feature. Our aim was to shed light on the frequency of HC in a cohort of patients with pSS and to evaluate which clinical and serological features are significantly associated with HC.

## Methods

### Patients

In total, the data of 333 patients with pSS who were admitted to Hebei General Hospital from September 2016 to March 2019 were retrospectively reviewed. All patients fulfilled the American-European Consensus Group 2002 revised classification criteria for pSS [18]. According to the presence of HC, our patients were divided into the study and control groups. The study was approved by the Research Ethics Committee of the Hebei General Hospital (NO. 2016070). Written informed consent was obtained from all participants.

### Procedures

General data and laboratory and clinical information at onset of patients with pSS were retrospectively reviewed, including the first presentation and systemic involvements. Clinical data, such as age at diagnosis, disease duration, oral and ocular dryness, and constitutional symptoms, as well as data on joint, pulmonary, kidney, vasculitis, skin, nervous, gastrointestinal tract, and endocrine involvement were collected. The levels of complements (C3 and C4), erythrocyte sedimentation rate, CRP, immunoglobulins (IgG, IgM, and IgA), RF, anti-Ro/SSA, and anti-La/SSB were retrieved from the case records. The laboratory tests were performed at the regular hospital laboratory. The disease activity was determined according to the 2010 ESSDAI [19, 20]. Disease activity was defined as low, moderate, and high, with scores of ≤4, 5–13, and ≥14, respectively [21]. Clinical and laboratory data were collected and according to a standard protocol. The duration of follow-up was defined as the interval from diagnosis until the date of the latest data collection (May 2021) or the date of SLE diagnosed or the date of death.

### Flow cytometry

Whole blood (5 ml) was collected from patients who were hospitalized before any treatment. Erythrocytes were lysed, and cells were harvested and washed twice and stained with a specific antibody at 4°C for 20 min. The following mAbs were used in this study (all from BD Pharmingen, San Diego, CA, USA): FITC-conjugated anti-CD3 mAb, phycoerythrin (PE)-conjugated anti-CD16/CD56 mAb, PE-cy7-conjugated anti-CD4 mAb, APC-conjugated anti-CD19 mAb, and APC-cy7-conjugated anti-CD8 mAb. Then, cells were subsequently washed and resuspended in phosphate-buffered saline prior to analysis. The stained cells were measured with a FACS Canto flow cytometer (Becton Dickinson, San Jose, CA, USA), and the data were analyzed using FlowJo Software (Treestar, Ashland, OR, USA).

### Statistical analysis

All data were analyzed using a standard statistical package (SPSS for Windows, version 25.0; IBM Corp., Armonk, NY, USA). Data are presented as means ± standard deviations, medians and interquartile ranges, or percentage frequencies, as appropriate. Group comparisons were conducted by Student’s t-test, Mann-Whitney’s U-test, or Fisher’s exact test, as appropriate. The correlations between variables were evaluated with Spearman’s rank correlation coefficient. The potential risk factors for HC were assessed by multiple logistic regression analysis, and the results are expressed as ORs and 95% CIs. Survival was estimated using the Kaplan-Meier method. The level of significance was set at *p* < 0.05.

## Results

### Prevalence of HC in patients with pSS

The study cohort consisted of 333 patients (310 women and 23 men). The HC rate was 25.23% (84/333). Among the 84 patients with pSS with HC, 78 (92.86%) were women and 6 (7.14%) were men. Those with HC were younger than those without (49.86 ± 15.49 vs. 55.30 ± 12.38 years; *p* = 0.004).

### Laboratory characteristics

As shown in Table 1, the patients with pSS with HC had striking significant differences between the two groups concerning the white blood cell (WBC) count (*p* < 0.001), in particular the neutrophil and lymphocyte counts (*p* = 0.003 and *p* = 0.005, respectively). The C-reactive protein (CRP) level was lower in patients with pSS-HC than in those with normal complement levels (*p* < 0.001), although the CRP level was approximately in a normal range. Additionally, the serum rheumatoid factor (RF) level (21.90 [10.6–158] vs. 16.55 [10.60–55.93]) was higher in the HC than in the normocomplementemia (NC) group, but the difference was not statistically significant (*p* = 0.44). Moreover, higher serum immunoglobulin G (IgG) levels were observed in the HC than in the NC group (*p* < 0.001). Further, higher presence of IgG (53.57% vs. 30.04%, *p* < 0.001) and anti-Ro52 positivity (71.43% vs. 56.22%, *p* = 0.01) were observed in patients with HC. Whereas, the positive rates of detecting antinuclear antibodies (ANA), ACA, anti-Ro/SSA, anti-La/SSB, and anti-RNP antibodies were not significantly different between the two groups. Besides, no significant difference in histologic evaluation of minor salivary gland, as a focus score ≥1, was observed between the two groups.

**Table 1:** Demographic and serological descriptors of patients with pSS with HC and NC

	Total group (*n* = 333)	HC group (*n* = 84)	NC group (*n* = 249)	*p*-value^#^
**Demographic features**				
Age at onset, years	54 ± 13.42	49.86 ± 15.49	55.30 ± 12.38	0.004
Disease duration, months	48 [12–120]	24 [6–84]	60 [12–120]	0.004
**Laboratory findings**				
White blood cell count (×10^9^/L)	4.96 [3.95–6.27]	4.52 [3.46–5.55]	5.12 [4.06–6.56]	0.001
Neutrophil counts (×10^9^/L)	2.94 [2.18–4.26]	2.71 [2.02–3.60]	3.11 [2.23–4.41]	0.003
Neutrophil proportion (%)	62.33 [53.66-68.47]	62.11 [53.62-68.00]	62.33 [53.64-68.79]	0.56
Lymphocyte counts (×10^9^/L)	1.51 [1.10–1.87]	1.35 [0.98–1.72]	1.57 [1.18–1.90]	0.005
Lymphocyte proportion (%)	29.93 [23.34-37.93]	29.59 [23.85-36.46]	30.42 [23.10-38.18]	0.90
Hemoglobin (×g/L)	122 [109.5–132]	118.5 [107–127]	122 [111–134]	0.007
Platelet counts (×10^9^/L)	224 [178–269]	199 [159.25–260]	228 [182–274]	0.006
ESR (mm/1 h)	19 [9–36]	15.5 [7–43]	19 [10–35]	0.27
CRP (mg/L)	3.3 [1.11–4.39]	1.27 [0.59–3.30]	3.3 [1.68–5.33]	<0.001
RF (IU/L)	17.10 [10.6–67.35]	21.90 [10.6–158]	16.55 [10.60–55.93]	0.44
IgG (g/L)	15.24 [12.4–19.93]	18.13 [13.16–25.50]	14.90 [12.20–18.13]	<0.001
IgA (g/L)	2.75 [1.92–3.63]	2.75 [1.80–3.90]	2.73 [2.02–3.55]	0.34
IgM (g/L)	1.17 [0.80–1.61]	1.12 [0.78–1.59]	1.18 [0.83–1.62]	0.48
Elevated ESR (n, %)	151/321, 47.04	36/80, 45.00	115/241, 47.72	0.67
Elevated CRP (n, %)	63/314, 20.06	8/76,10.53	55/238, 23.11	0.02
Hyper-IgG (n, %)	118/327, 36.09	45/84, 53.57	73/243, 30.04	<0.001
RF (+) (n, %) *	150/311, 48.23	40/78, 51.28	110/233, 47.21	0.45
ANA (+) (n, %) **	263, 78.98	66, 78.57	197, 79.12	0.92
Anti-RNP (+) (n, %)	39, 11.71	12, 14.29	27, 10.84	0.40
Anti-Ro52 (+) (n, %)	200, 60.06	60, 71.43	140, 56.22	0.01
Anti-Ro/SSA (+) (n, %)	187, 56.16	54, 64.29	133, 53.41	0.08
Anti-La/SSB (+) (n, %)	79, 23.72	26, 30.95	53, 21.29	0.07
ACA (+) (n, %)	45, 13.51	11, 13.10	34, 13.65	0.90
Pathological MSG with focus score ≥1 (n, %)	309/323, 95.67	78/80, 97.50	231/243, 95.06	0.53

*pSS*, primary Sjögren’s syndrome; *RF*, rheumatoid factor; *ANA*, antinuclear antibodies; *MSG*, minor salivary gland; *ACA*, anti-centromere antibodies; *ESR*, erythrocyte sedimentation rate; *IgM*, immunoglobulin M; *IgA*, immunoglobulin A; *IgG*, immunoglobulin G; *CRP*, C-reactive protein; *NC*, normal complement; *HC*, hypocomplementemia

^**#**^*p* value: HC group vs. NC group; *Positive RF > 20 IU/mL; **positive for ANA titers ≥ 1:320

### Complement and autoantibodies

In the pSS cohort, the levels of serum C3 and C4 in anti-Ro/SSA-positive were 1.02 (0.89–1.20) g/L and 0.18 (0.15–0.23) g/L, which were significantly lower than anti-Ro/SSA-negative patients (1.11 (0.96–1.22) g/L, *p* = 0.006; 0.20 (0.17–0.24) g/L, *p* = 0.004; respectively). When further stratifying for the presence or absence of anti-La/SSB antibodies, the level of serum C4 in anti-La/SSB-positive was significantly lower than anti-La/SSB-negative patients (0.16 (0.13–0.23) g/L vs. 0.20 (0.17–0.24) g/L, *p* = 0.001). In addition, no significant difference in complement C3 was observed between anti-La/SSB-positive and anti-La/SSB-negative patients at baseline. Whereas, at baseline, no significant differences in complement C3 and C4 were observed between RF-positive and RF-negative nor ANA-positive and ANA-negative patients (Supplementary Fig. 1).

### Clinical manifestations

**Fig. 1:**
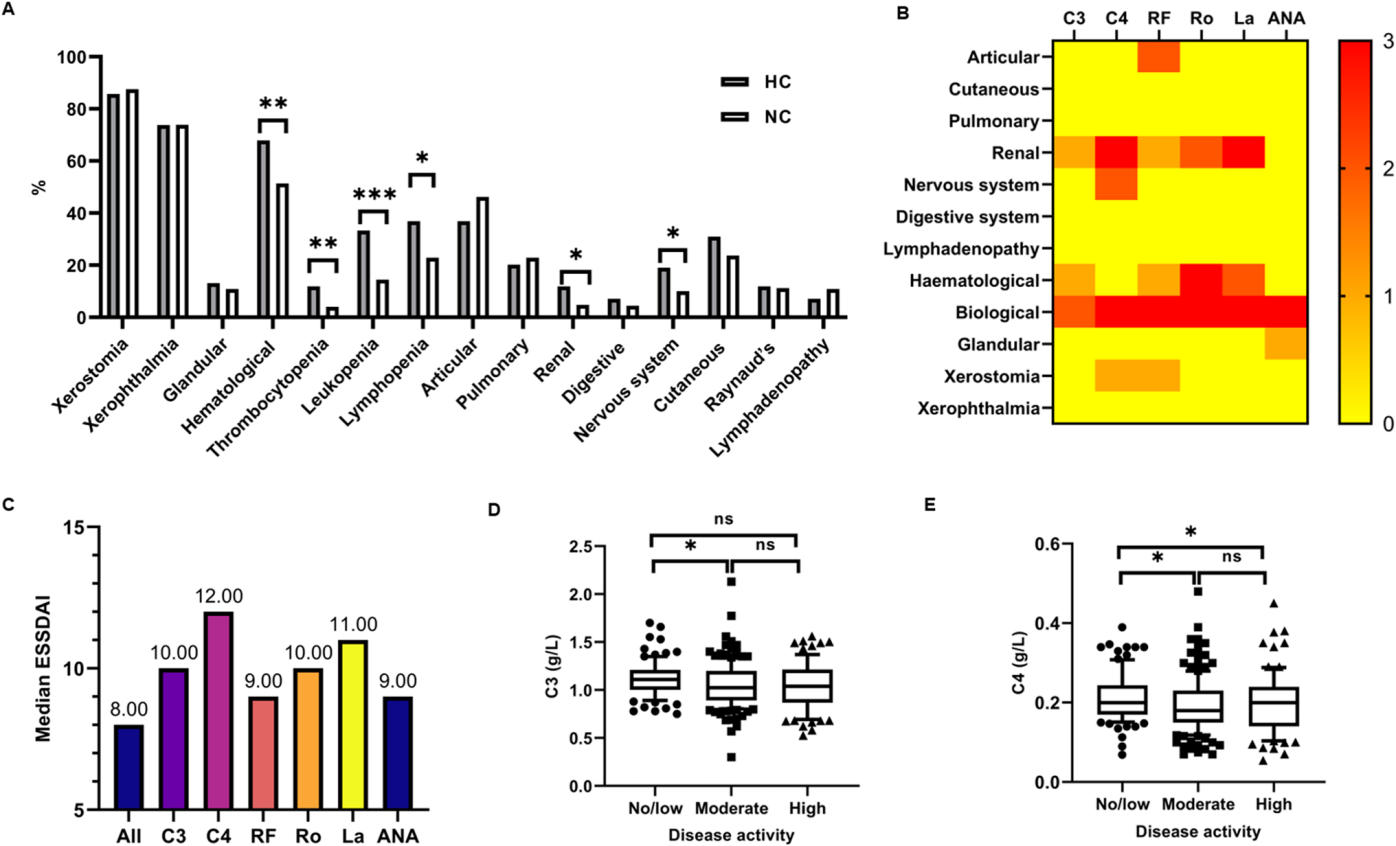
Clinical manifestations and disease activity of pSS patients according to the level of complement at diagnosis **A**. Systemic involvement of the two groups of patients according to the level of complement at pSS diagnosis. **B**. Heat map of the main statistically-significant associations between immunological markers and disease phenotype. **C**. Median ESSDAI according to each immunological marker. **D**, **E**. Baseline C3 and C4 levels according to ESSDAI disease activity. *p < 0.05, **p < 0.01, ***p < 0.001

In our study, the patients with pSS with HC more commonly exhibited hematological involvement (67.86% vs. 51.41%; *p* = 0.009), thrombocytopenia (11.90% vs. 4.02%; *p* = 0.009), leukopenia (33.33% vs. 14.46%; *p* < 0.001) and lymphopenia (36.90% vs. 22.89%; *p* = 0.01) at pSS diagnosis (Fig. 1 and Supplementary Table 1). Compared to patients with pSS-NC, the patients in the HC group presented higher renal (11.90% vs. 4.82%, *p* = 0.02) and nervous system involvement (19.05% vs. 10.04%, *p* = 0.03). There were no significant differences in the classic symptoms of sicca syndrome, such as xerostomia, xerophthalmia, and salivary gland enlargement (*p* = 0.66, *p* = 0.99, and *p* = 0.58, respectively). Meanwhile, although the difference was not statistically significant, the incidence of cutaneous involvement was higher (*p* = 0.19), whereas the frequencies of arthritis, pulmonary involvement, and lymphatic system involvement were lower (*p* = 0.14, *p* = 0.61, and *p* = 0.33, respectively) in patients with pSS with HC than in those with NC.

We then evaluated associations between different immunological markers and disease phenotype. It was not the same as the system affected by other immune markers, such as RF, anti-Ro/SSA, anti-La-SSB and ANA. Low C3 was detected in nearly a quarter of our patients, who showed a specific phenotype consisting of a high median ESSDAI score, and a high frequency of systemic activity in the renal, hematological and biological domains. C4-HC patients also showed a specific phenotype consisting of a higher frequency of abnormal diagnostic tests for xerostomia, a much higher median ESSDAI score, and a high frequency of systemic activity in the renal, nervous, and biological domains. At the baseline, all variables were statistically significant independent factors in at least two domains.

The baseline mean ESSDAI was significantly higher in the HC patients compared with the NC patients (ESSDAI 10.5 [6–16] vs. 7 [3–13]; *p* = 0.006). Moreover, statistically significant differences were observed in baseline complement C3 (*p* = 0.03) and C4 (*p* = 0.04) levels explored by one-way ANOVA between patients who had no/low disease activity (defined as ESSDAI score) compared with those who had moderate or high disease activity (Fig. 1).

### Lymphocyte profile in hypocomplementemia patients

pSS patients with HC had a significantly lower number of circulating lymphocytes compared with normal complement group (1.35 [0.98–1.72] × 10^9^/L vs. 1.57 [1.18–1.90] × 10^9^/L, *p* = 0.005). Further, we assessed immunophenotyping of pSS patients with or without hypocomplementemia by flow cytometry to investigate lymphocyte profile. As shown in Supplementary Fig. 1, Supplementary Fig. 3, and Supplementary Table 2, in HC patients, a significance decreases in absolute numbers of CD3+ T cells (919.89 [669.03–1153.09]/μl vs. 1100.67 [789.87–1331.01]/ul, *p* = 0.02), CD3/CD4^+^T cells (434.52 [308.82–632.49]/μl vs. 579.54 [399.76–779.83]/ul, *p* = 0.001), CD16/CD56+ NK cells (135.33 [78.99–221.78]/μl vs. 190.94 [126.82–311.10]/μl, *p* = 0.001) was found. Whereas the absolute numbers of CD3/CD8^+^ T cells and CD19^+^ B cells were comparable between the two groups (*p* = 0.55 and *p* = 0.54). At the meantime, CD3/CD4^+^ T lymphocyte percentage and CD16/CD56^+^ NK lymphocyte percentage were significantly reduced. Whereas, there were no significant difference in CD3^+^ T lymphocyte percentage, CD3/CD8^+^ T lymphocyte percentage, or CD19^+^ B lymphocyte percentage between the two groups.

### Correlations between serum complement levels and clinical characteristics, lymphocyte profile

**Fig. 2:**
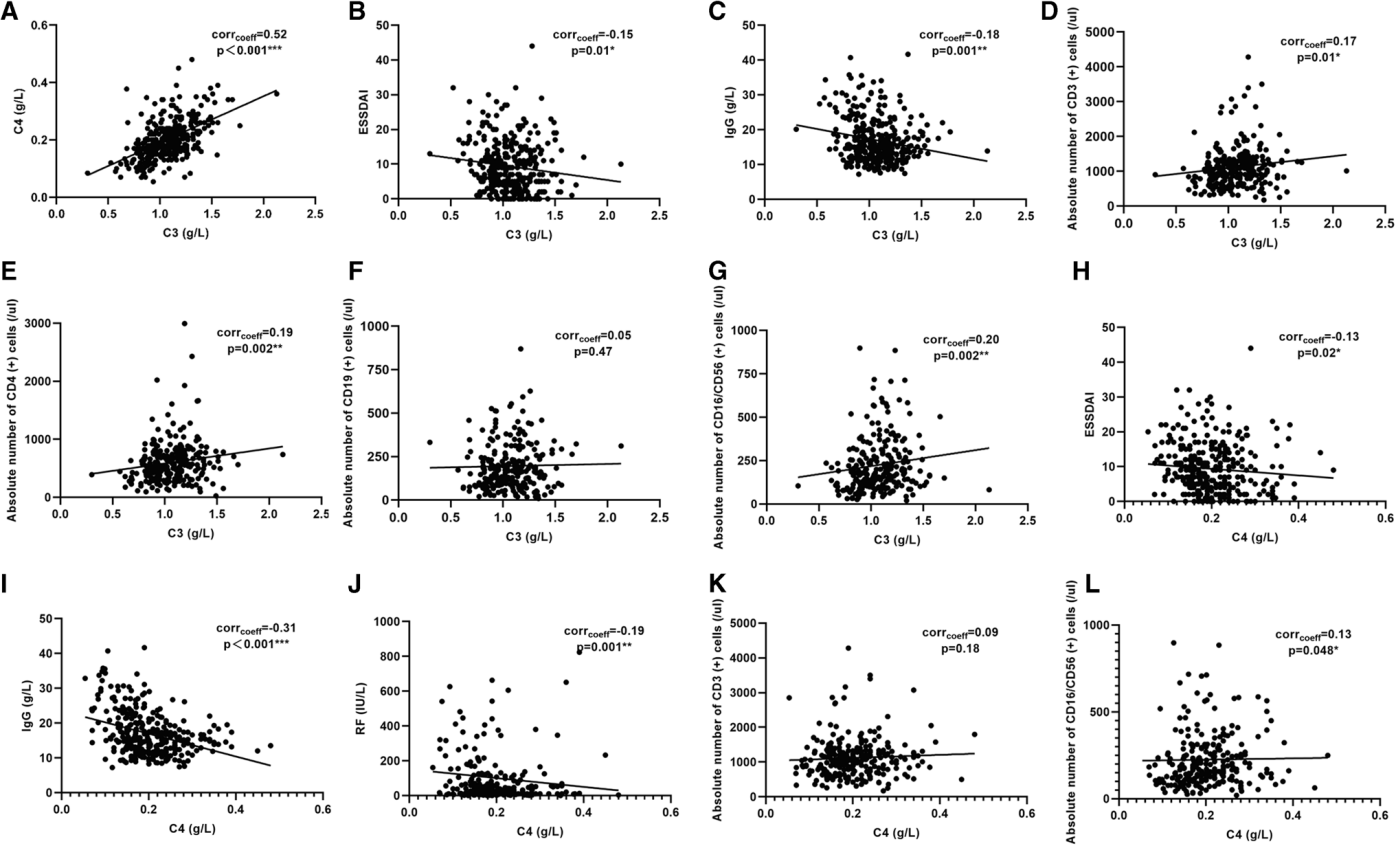
Correlations between baseline serum complement levels and clinical characteristics in pSS patients. **A**–**C** Correlations between baseline serum C3 level and clinical characteristics. **D**–**G** Correlations between baseline serum C3 level and lymphocyte profile. **H**–**J** Correlations between serum C4 level and clinical characteristics. **K**–**L** Correlations between baseline serum C4 level and lymphocyte profile

Spearman correlation coefficient analysis was performed to investigate the association between serum complement level and ESSDAI, laboratory parameters and lymphocyte profile in total patients at baseline. As shown in Fig. 2, serums C3 showed a significant positively correlation with C4 (r = 0.52, *p* < 0.001). The level of serum C3 was negatively correlated with ESSDAI (*r* = −0.15, *p* = 0.01), IgG (*r* = −0.18, *p* = 0.001), whereas positively correlated with ESR (*r* = 0.17, *p* = 0.02), CRP (*r* = 0.38, *p* < 0.001), lymphocyte (*r* = 0.19, *p* < 0.001), platelet (*r* = 0.29, *p* < 0.001), and peripheral lymphocyte subsets such as absolute numbers of CD3^+^ T cells (*r* = 0.17, *p* = 0.01), CD4^+^ T cells (*r* = 0.19, *p* = 0.002) and CD16/CD56^+^ N K cells (*r* = 0.20, *p* = 0.002). Similarly, serum C4 level was negatively correlated with ESSDAI (*r* = −0.13, *p* = 0.02), IgG (*r* = −0.31, *p* < 0.001), and RF (*r* = −0.19, *p* = 0.001), whereas positively correlated with CRP (*r* = 0.32, *p* < 0.001), platelet (*r* = 0.19, *p* = 0.001), and as well as absolute numbers of CD16/CD56+ NK cells (*r* = 0.13, *p* = 0.048).

### Risk factors for HC in patients with pSS

Multivariate analysis confirmed that leukopenia (odds ratio [OR] = 2.23, 95% confidence interval [CI] 1.18–4.23, *p* = 0.01) and hyper-IgG (OR = 2.13, 95% CI 1.22–3.72, *p* = 0.01) were independent predictors of HC in patients with pSS (Table 2).

**Table 2:** Multivariate analysis of features predicting HC in patients with pSS

	p-value	OR	95% CI
Leukopenia	0.01	2.23	1.18–4.23
Anemia	0.07	1.69	0.96–2.99
Elevated CRP	0.02	0.36	0.16–0.82
Hyper-IgG	0.01	2.13	1.22–3.72

*CI*, confidence interval; *OR*, odds ratio; *pSS*; primary Sjögren’s syndrome; IgG, immunoglobulin G; *CRP*, C-reactive protein

### Therapeutic regimens and follow-up examinations

All pSS patients were treated, including glucocorticoids (GCs) alone, GCs combined with immunosuppressant agents (GCs plus IM), GCs combined with rituximab (RTX) or hydroxychloroquine alone. In the pSS-HC group, 11 (13.10%) patients received GC monotherapy. Thirty-five (41.67%) patients received hydroxychloroquine alone. One (1.19%) patient received GCs plus RTX. Others were treated with GCs plus IM or IM monotherapy, including leflunomide (*n* = 15, 17.86%), mycophenolate mofetil (MMF) (*n* = 4, 4.76%), cyclosporin A (*n* = 5, 5.95%), iguratimod (*n* = 3, 3.57%), and cyclophosphamide (*n* = 1, 1.19%). While GCs-based therapies (GCs alone or in combination with IM/RTX) were used more common in HC group (29.76% vs 21.29%), the difference was not statistically significant (*p* = 0.11). When compared with NC group, more HC patients received MMF (4.76% vs. 0.80%, *p* = 0.04) and cyclosporin A (5.95% vs. 1.20%, *p* = 0.03).

**Fig. 3:**
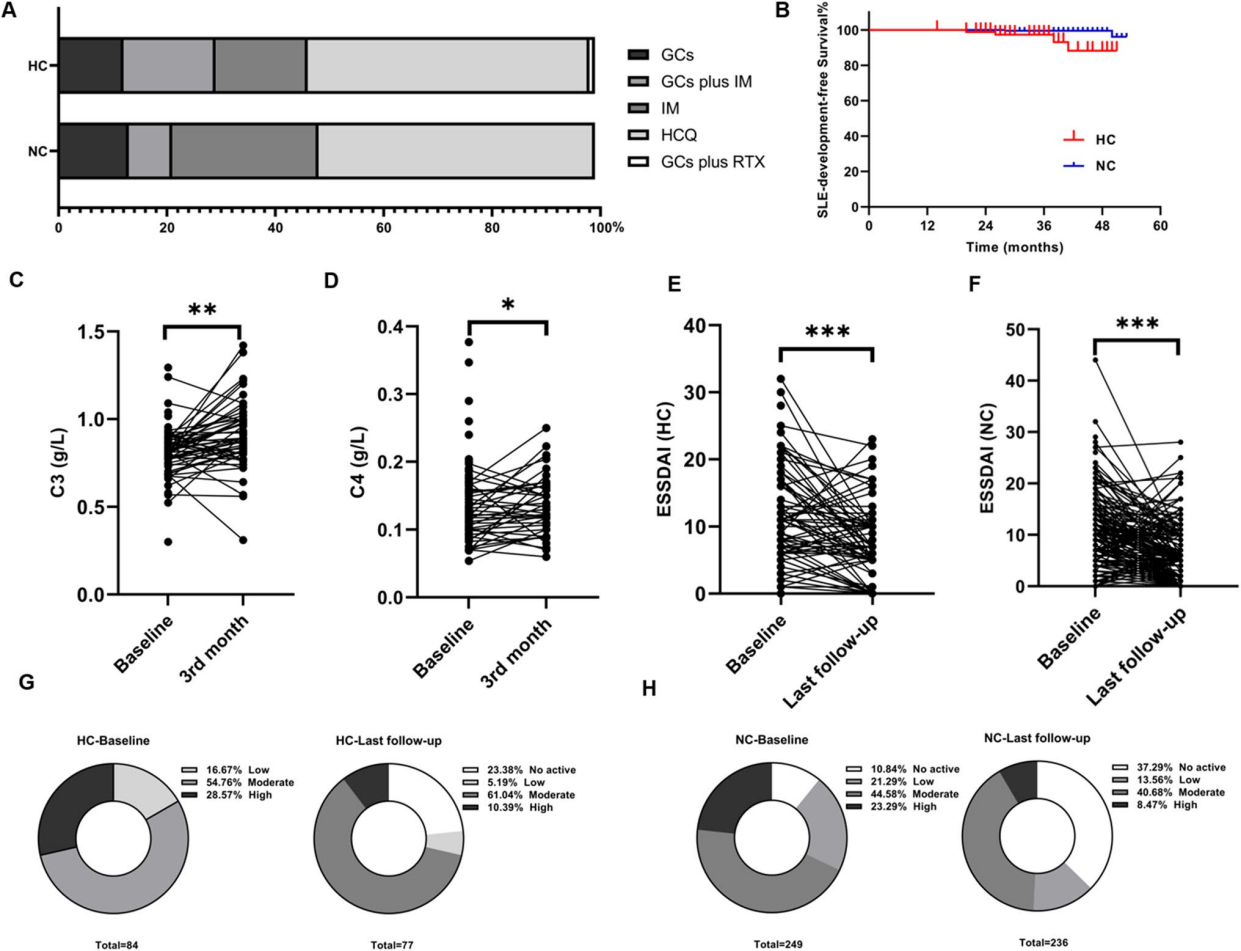
Comparison of treatment regimens and therapeutic outcomes of pSS patients in HC and NC group. **A**. The first-line therapies for HC group and NC group. **B**. Kaplan-Meier survival analysis suggested significant difference of SLE-development-free survival between two groups (*p* = 0.04). Graphs show the level of serum C3 (**C**) and C4 (**D**) at baseline and 3 months’ follow-up. Graphs show the changes of ESSDAI during the 48 months of follow-up in HC group (**E**) and NC group (**F**). Graphs show the improvements of disease activity at baseline and at the last follow-up visit in pSS patients with HC (**G**) and with NC (H). *p < 0.05, **p < 0.01, ***p < 0.001

The level of serum C3 and C4 significantly increased after treatment (*p* < 0.05), but still not to the normal range at the third month. During a similar follow-up time (34.03 ± 10.47 months vs. 32.78 ± 12.02 months, *p* = 0.28), more pSS patients developed SLE in the HC group (*n* = 4, 4.76%) than NC group (*n* = 2, 0.80%, *p* = 0.04). With a mean follow-up of 34.50 ± 10.19 months (range 24–50 months) after diagnosis of pSS, these patients developed SLE. Further, one patient (1.19%) in HC group died, which was comparable to NC group (*n* = 3, 1.20%, *p* = 0.99). The ESSDAI in HC patients was still higher than that in NC patients (6 (1.5–10) vs 4 (0–7)); *p* = 0.001) at the last follow-up visit, which was significantly lower compared to baseline at diagnosis (10.5 (6–16), *p* < 0.001). In addition, disease activity was low in 14 (16.67%) patients, moderate in 46 (54.76%), and high in 24 (28.57%) at baseline in HC patients. After therapy, disease activity was no activity in 18 (23.38%) patients, low in 4 (5.19%), moderate in 47 (61.04%), and high in 8 (10.39%) in HC patients. Improvement in the ESSDAI at the last follow-up visit, compared to that at baseline, was also observed in the NC group (4 (0–7) vs. 7 (3–13); *p* < 0.001), and disease activity was no activity in 88 (37.29%) patients, low in 32 (13.56%), moderate in 96 (40.68%), and high in 20 (8.47%) (Fig. 3).

## Discussion

In this study, for the first time, we confirmed the association of HC with the phenotype of the disease not only at diagnosis but also at the end of follow-up in the large cohort of Chinese pSS patients. Complement values were measured in 333 pSS patients and were low in 84 (25.23%). HC had an independent association with the main clinical and immunological features, including a lower mean age at diagnosis, a higher serum IgG level, a higher ESSDAI score both at diagnosis and at the end of follow-up, and a higher frequency of activity in hematological, renal, and nervous system. To the best of our knowledge, this study evaluated the lymphocyte profile in pSS-HC patients for the first time, and profound CD16/CD56+ NK lymphopenia and decreased numbers of CD4+ T-cell cells were the most distinguishing features of the HC group. As a marker of pSS activity, the measurement of C3 and C4 levels was recommended to evaluate and monitor the condition of patients with pSS. In addition, multivariate analysis identified leukopenia and elevated IgG as risk factors for HC in patients with pSS.

The prevalence of low C3 and C4 levels was found to be 22.82% and 8.71% in this study, which was similar to previous studies. Especially, Baldini et al. [22] detected low C3 and C4 levels in 15.3% and 11.1% of 1115 patients, respectively. Jordán-González et al. [23] found low C3 and C4 levels in 9.57% and 13.83% of 94 patients, respectively, while Lin et al. [24] reported low C3 and C4 levels in 14.4% and 16.5% of their participants, respectively. The different ethnicity, cohort size, disease severity degrees, and cut-off levels of the complement assays used may be attributed to the different prevalence of HC observed in these studies.

In a large international multicenter study and another study including 921 Spanish pSS patients, reduced C3 and C4 complement fractions at the pSS diagnosis were observed and these findings were associated with disease activity [25, 26]. Besides, a systematic review and meta-analysis were performed and found that low C3 [RR 2.14 (95% CI 1.38, 3.32)] and C4 levels [RR 3.08 (95% CI 2.14, 4.42)] were consistently associated with higher mortality risk [27]. These results suggested that complement consumption may contribute to the pSS etiopathogenesis. We assessed for the first time that HC not only correlates with disease activity at diagnosis, but also plays a central role in predicting outcome as prognostic marker. The current study is, to the best of our knowledge, the first to describe the detailed characteristics of HC in pSS patients not only at diagnosis but also at the end of follow-up, and attempts to find the possible pathogenic mechanisms.

Several previous studies have explored the potential role for abnormal complement activation in pSS. Especially, Zadura et al. found that with the widespread autoantibody production, the C4BP levels decreased in parallel with the C3 and C4 levels in active patients with pSS, which suggested that disturbed complement regulation may contribute to pSS pathogenicity [28]. Interestingly, Sudzius et al. reported that the serum C4d level was significantly lower in anti-SSA/SSB Ab seropositive than in seronegative patients with pSS and was also correlated with the C4 and anti-SSB Ab levels and the κ/λ ratio. They proposed that the C4d level can be an appropriate marker of complement activation in patients with pSS with Abs [29]. Evans et al. reported the presence of positive staining for C9 around the tubular basement membranes in patients with pSS-TIN [30]. Moreover, Xia et al. performed a retrospective study and investigated the prevalence and localization of C4d deposits in renal biopsy tissues of patients with pSS. They found that glomerular C4d deposition was observed in all patients with pSS-related membranous nephropathy (MN) and suggested a role for the mannan-binding lectin pathway of complement activation in patients with pSS-related MN [31]. Furthermore, our lymphocyte profile studies revealed that, for the first time, significant decreases in absolute numbers of CD16/CD56+ NK cells were found in HC patients, and had a positive correlation with both C3 and C4, which might enforce the concept that complement activation affects the function of NK cells [32]. With the evidence of the complement system in vivo activation, these results suggested that complement consumption affects pSS pathophysiology. Therefore, the presence of HC in patients with pSS may signify the presence of disease activity. This is consistent with our findings, which demonstrated a significant higher ESSDAI in patients with pSS-HC.

HC had no influence on the glandular disease expression, but played an important role in driving a multi-systemic phenotype with statistically significant higher frequencies, including renal, nervous, and hematological involvement in our study. In recent years, there has been an increasing interest in renal diseases associated with pSS. Specifically, scientists are interested in whether the reduction of complement in pSS patients is related to kidney involvement. In our study, HC was more commonly found in patients with renal disease than in those in other groups, which was in line with previous findings [33, 34]. However, this finding was contradicted by other studies. Especially, Zhao et al. found that hypokalemic paralysis, assumed to be a surrogate of renal damage, did not show any association with low complement [35]. Moreover, Yang et al. published a retrospective study, which included 103 patients with pSS who had undergone kidney biopsy. Interestingly, no significant differences were observed between patients with and without renal disease [36]. Combined with the pathological findings, it was suggested that complement activation may have contributed to Sjogren’s nephritis development.

Nervous system involvement, ranging from the peripheral nervous system to the CNS, is known to be multifaceted and possibly underestimated in pSS. Neurological disease might have arose many years before pSS diagnosis and was found to contribute to damage in patients with pSS. Our study is one of the first to report that nervous system involvement correlates with HC in patients with pSS. Besides, we found that there was a relationship between the incidences of nervous system involvement and HC, which is consistent with the findings by Ye et al., who found that patients with pSS with neurological involvement showed reduced C3 levels (*p* < 0.05). Additionally, low C3 level was found to be a potential risk factor for neurological involvement in younger patients with pSS [37]. Moreover, arising data supported that the complement system may facilitate various neuroinflammation activities [38, 39]. Because of the evidence indicating that the complement levels play key roles in driving multiple nervous pathologies, the complement system may be a potential therapeutic target for treating brain injury in patients with pSS [40].

What’s more, HC was strongly associated with disease prognosis and outcomes. It is well known that the complement system plays a pivotal role in SLE development, and previous data reported that patients with pSS with low complement levels are more likely to develop SLE. In a retrospective study including 55 patients with Sjogren’s syndrome-onset SLE (SS/SLE), Yunjiao et al. revealed that these patients showed a significantly higher frequency of low complement levels (C3, 54.5% vs. 12.7%; C4, 41.8% vs. 7.3%, *p* = 0.000). Additionally, low C3 and C4 levels were independent risk factors of SS/SLE development (low C3 levels, RR = 9.659, *p* = 0.000; low C4 levels, RR = 6.035, *p* = 0.007) [41]. Our study showed that pSS with HC can precede SLE by several years, confirming previously published reports of patients with long-standing pSS who eventually developed a systemic disorder that fulfilled the criteria for SLE [42]. The coexistence of SS and SLE was first demonstrated in 1959 [43] and has since been demonstrated by multiple studies. Some studies have shown that pSS with extraglandular manifestations can precede SLE by several years and reports suggest that 3.7–19.6% of SLE patients are accompanied by SS. Zufferey et al. found that 4 (7.5%) SS patients developed SLE after a long-term follow-up (range 8–18 years) of 55 SS patients [44]. Manoussakis et al. evaluated 283 SLE patients and found that SS was identified in 26 SLE patients (9.2%), of which the SS preceded the development of lupus in 18 of them (69.2%) [45]. Compared with SLE alone, this combined disease of SS-SLE has distinct features with relatively less major internal organ involvement but has more specific immunologic profile and favorable clinical outcome [46]. From this point of view, Menelaos N. Manoussakis et al. suggested that, SLE-SS might be considered a specific subgroup of pSS, as disease manifestations in patients with SLE-SS likely represented a continuum ranging from the classic presentation of pSS with minimal lupus manifestations to the more recognizable lupus process with sicca features [45]. The exact cause of emerging SLE in patients with pSS has not been well known yet. They share several possible underlying etiopathogenic aspects, including numerous genetic factors, epigenetic, environmental, and hormonal factors [47–49]. Thus, careful investigation of SLE in patients with pSS diagnosis is recommended. However, pSS and SLE cannot be differentiated definitely. One of the main challenges faced by studies of pSS is that several of the systemic features, including lymphopenia, central and peripheral nervous system manifestations, also occur in SLE patients. In addition, these two entities share several immunological features, including the presence of anti-SSA and/or SSB antibodies. Therefore, it is difficult to distinguish systemic pSS from SLE, especially when antibodies against double-stranded DNA or Sm, which are specific for SLE and very rare in pSS, are negative. More clinical research is needed to establish definitive diagnosis criterion to distinguish these two important conditions.

Although the mechanisms underlying lymphoma pathogenesis in patients with pSS have not been well identified, low C3/C4 levels may help improve the survival of autoreactive B-cells in such patients and further, through mutations, increase the risk of lymphoid malignancy occurrence [50, 51]. HC was thought to be associated with lymphoma development and worse prognosis in patients with pSS [17, 22, 52]. In our study, it is noteworthy that such patients with HC usually presented hypergammaglobulinemia, anemia, leukopenia, and, especially, lymphocytopenia at the time of pSS diagnosis, indicating that these conditions are strong predictors of lymphoma development. In accordance with previous reports, we confirmed the preponderance of high risk of lymphoma development in the HC group.

The limitations of this study include several aspects. First, the study has a retrospective design. Second, we only tested some patients with suspected cryoglobulinemia, and the results were all negative, so cryoglobulin was not routinely checked in all our patients. This might be partly because cryoglobulinemia was relatively rare in Chinese pSS patients [53], when compared with European studies [22, 54, 55]. However, we conducted a detailed systematic evaluation of the enrolled patients, especially the ESSDAI domains that might be related to cryoglobulinemia, such as the kidney, mucous membrane, and nervous system. What’s more, there were no hepatitis C and lymphoma patients in our group. To address this issue, a future study analyzing the predictive value of cryoglobulinemia in Chinese patients with pSS is required. Third, in this study, potential clinical factors in estimating the prognosis, such as longer follow-up changes in C3/C4 levels, were not well evaluated. Further prospective studies with a larger number of long-term follow-up patients with pSS are needed.

In conclusion, we confirmed that HC was associated with specific clinical and immunological features in patients with pSS. It is noteworthy that such patients with HC usually presented hypergammaglobulinemia, anemia, leukopenia, and, especially, CD16/CD56 (+) NK lymphocytopenia at the time of pSS diagnosis, thus highlighting the increased risk of lymphoproliferative disease development. In addition, complement level was a potential marker of disease activity in pSS. Furthermore, this study revealed that HC might help in the early identification of patients with pSS who need to receive more aggressive treatments. Thus, complement detection in pSS is clinically useful, especially for the diagnosis and prognosis. However, further large-scale prospective studies are needed to confirm these findings.

## Supplementary information

ESM 1 (PNG 458 kb)

High Resolution Image (TIF 296 kb)

ESM 2 (PNG 540 kb)

High Resolution Image (TIF 305 kb)

ESM 3 (DOCX 251 kb)

## Data Availability

The datasets used or analyzed during the current study are available from the corresponding author on reasonable request.
