# Supplementary figures and images for: Hypocomplementemia in primary Sjogren’s syndrome: association with serological, clinical features, and outcome
Source: Clin Rheumatol. 2022 Mar 29;41(7):2091–102. doi: 10.1007/s10067-022-06135-w (PMC9187545; doi:10.1007/s10067-022-06135-w)

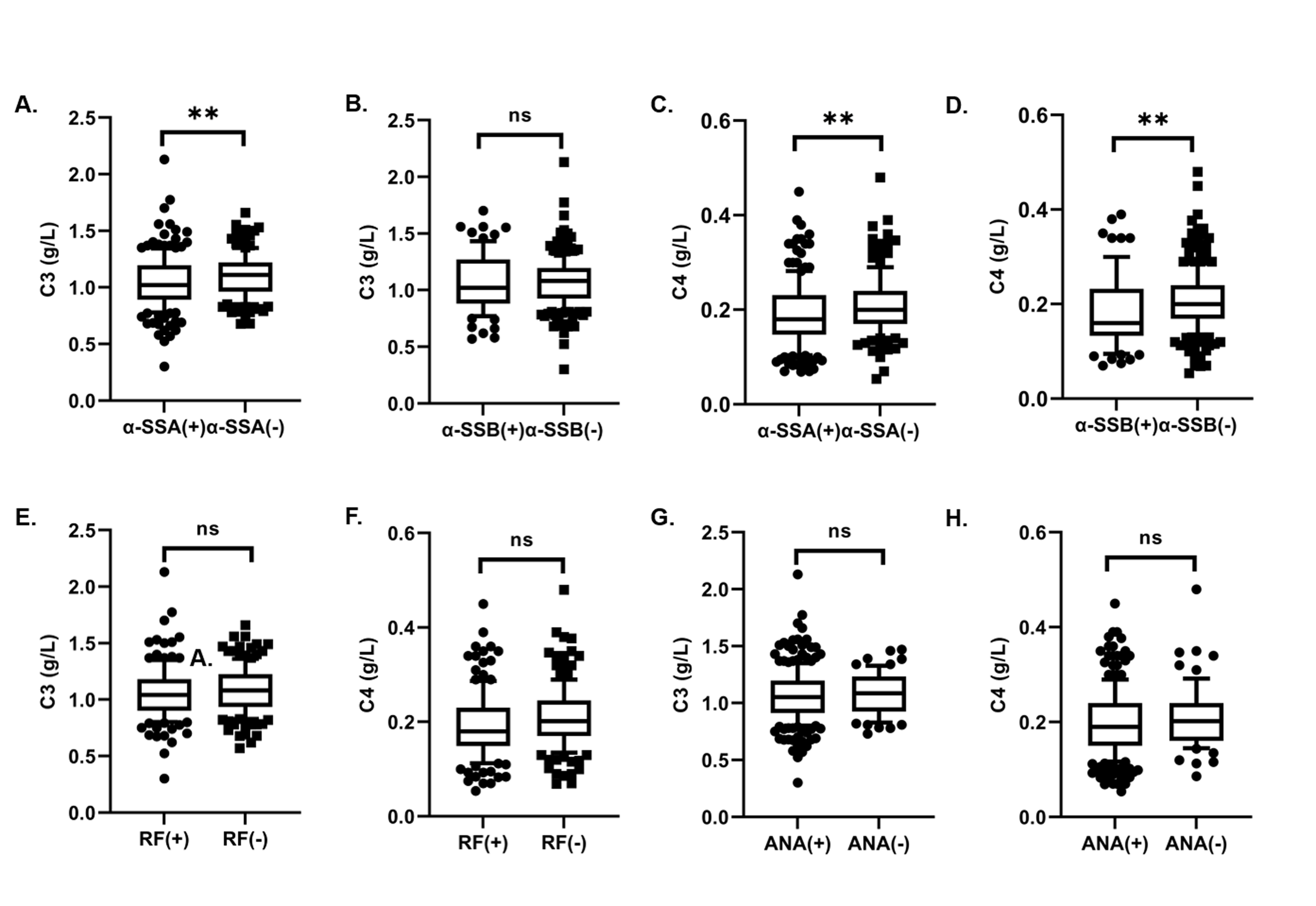

Supplement: Supplementary file 1 — (PNG 458 kb) [file 10067_2022_6135_Fig4_ESM.png]

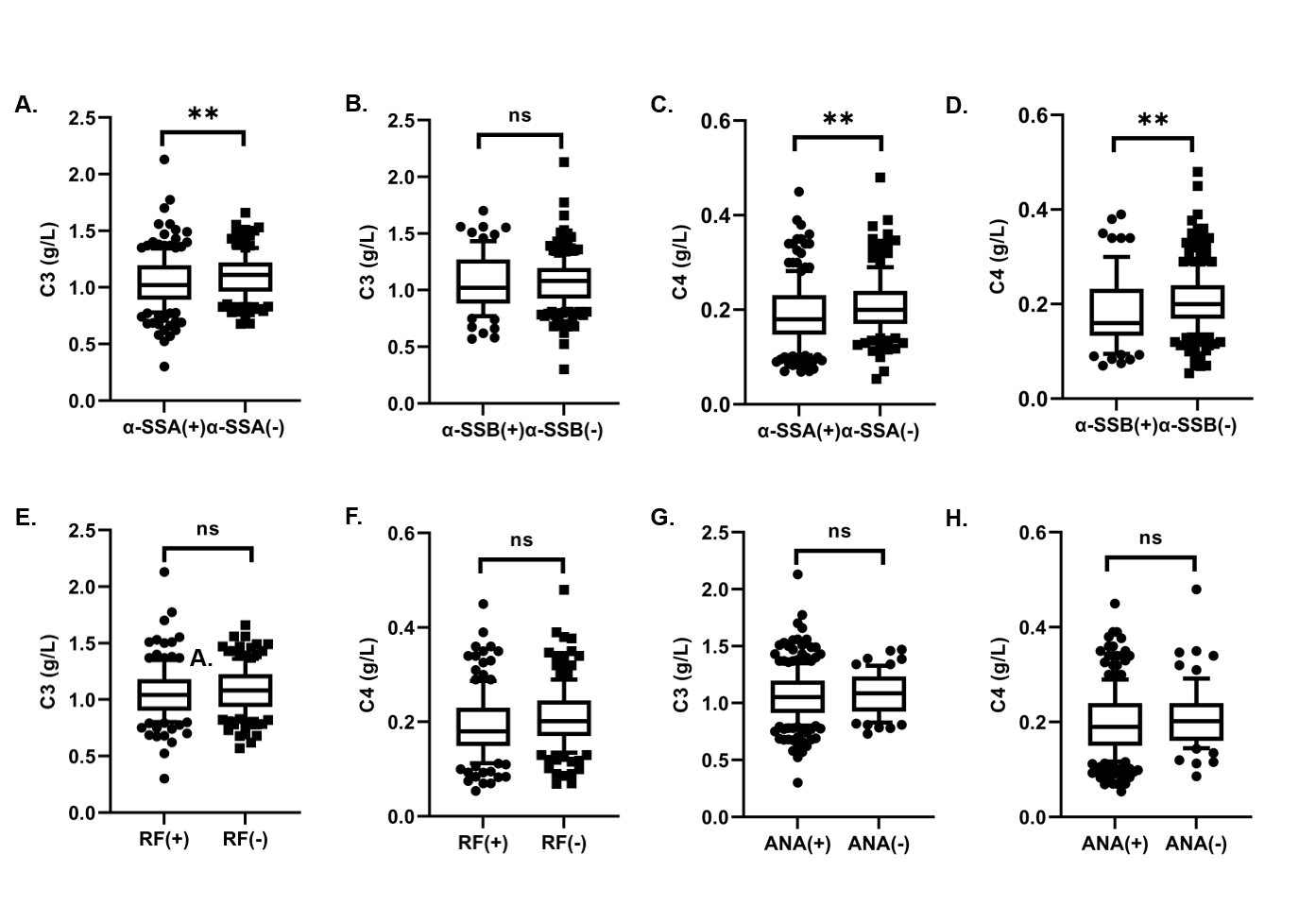

Supplement: Supplementary file 2 — High Resolution Image (TIF 296 kb) [file 10067_2022_6135_MOESM1_ESM.tif]

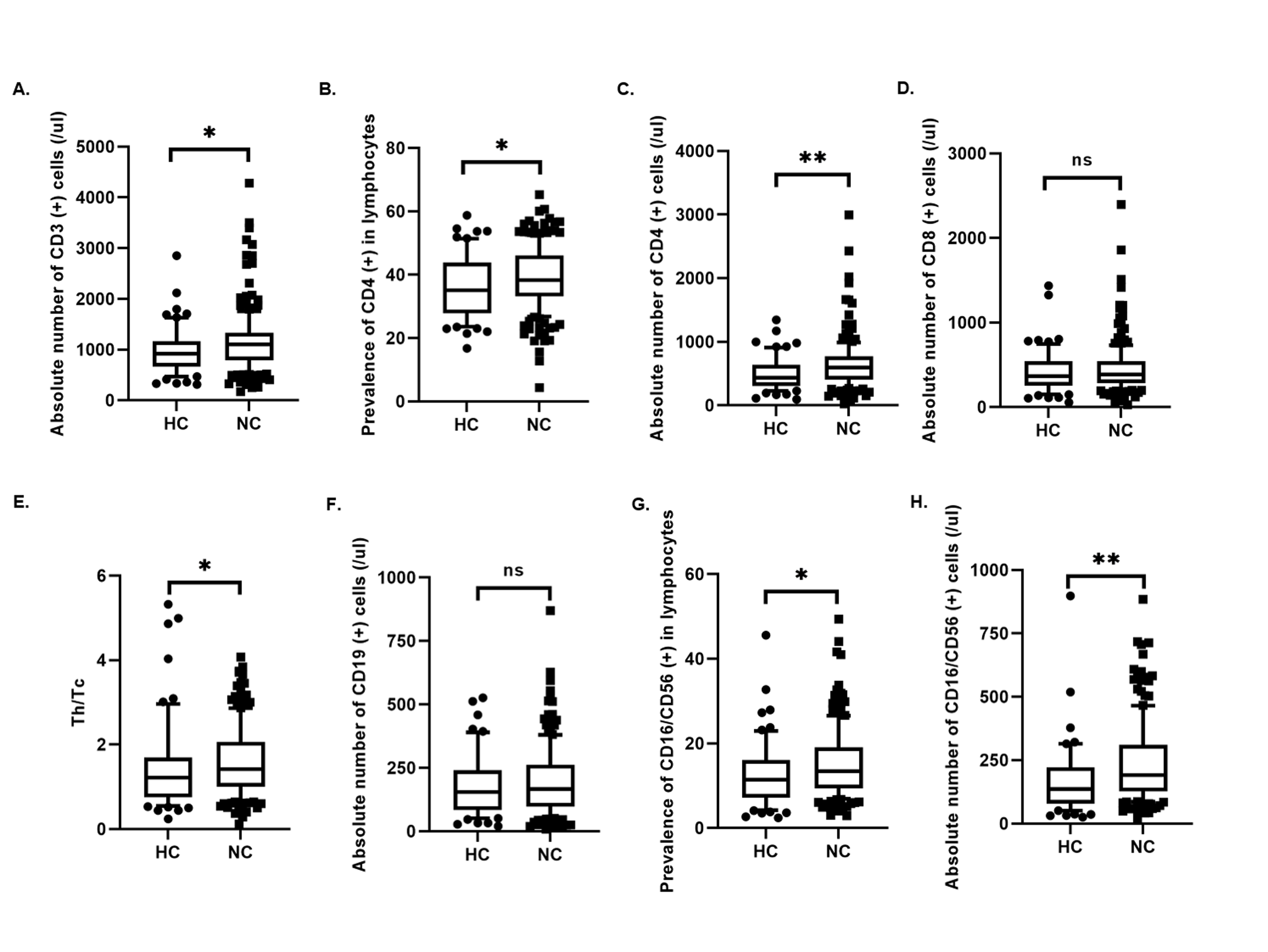

Supplement: Supplementary file 3 — (PNG 540 kb) [file 10067_2022_6135_Fig5_ESM.png]

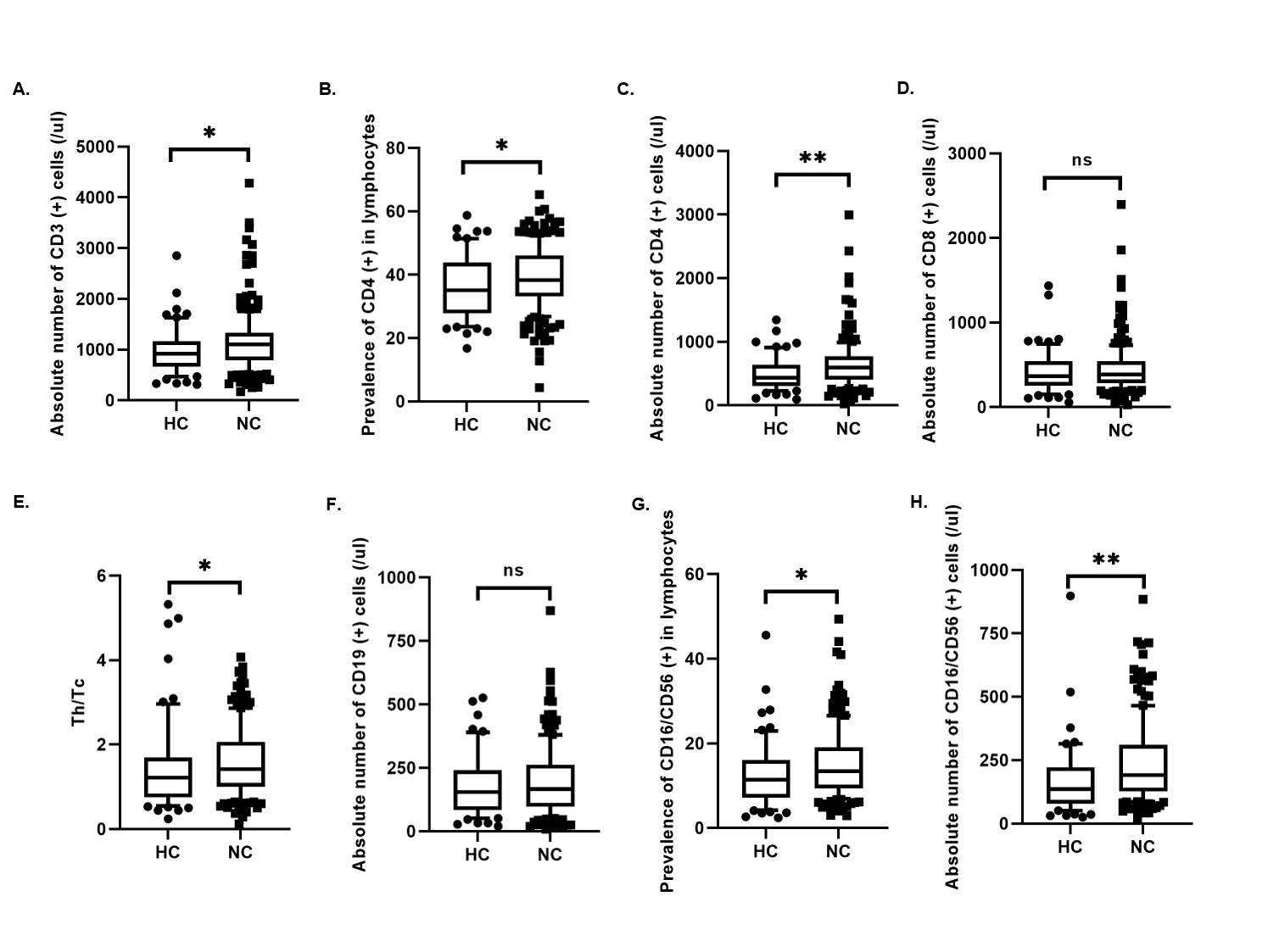

Supplement: Supplementary file 4 — High Resolution Image (TIF 305 kb) [file 10067_2022_6135_MOESM2_ESM.tif]
