# Supplementary material for: Hypocomplementemia in primary Sjogren’s syndrome: association with serological, clinical features, and outcome
Source: Clin Rheumatol. 2022 Mar 29;41(7):2091–102. doi: 10.1007/s10067-022-06135-w (PMC9187545; doi:10.1007/s10067-022-06135-w)

**Sup-Table 1.** Comparisons of systemic involvement between patients with pSS with low (HC) and normal (NC) complement levels

| **Variables (n/%)** | **HC group**  **(n = 84)** | **NC group**  **(n = 249)** | **p-value** |
| --- | --- | --- | --- |
| Xerostomia | 72, 85.71 | 218, 87.55 | 0.66 |
| Xerophthalmia | 62, 73.81 | 184, 73.90 | 0.99 |
| Salivary gland enlargement | 11, 13.10 | 27, 10.84 | 0.58 |
| Hematological involvement | 57, 67.86 | 128, 51.41 | 0.009 |
| Thrombocytopenia | 10, 11.90 | 10, 4.02 | 0.009 |
| Leukopenia | 28, 33.33 | 36, 14.46 | <0.001 |
| Lymphopenia | 31, 36.90 | 57, 22.89 | 0.01 |
| Arthritis | 31, 36.90 | 115, 46.18 | 0.14 |
| Pulmonary involvement | 17, 20.24 | 57, 22.89 | 0.61 |
| Renal involvement | 10, 11.90 | 12, 4.82 | 0.02 |
| Digestive involvement | 6, 7.14 | 11, 4.42 | 0.40 |
| Nervous system involvement | 16, 19.05 | 25, 10.04 | 0.03 |
| Cutaneous involvement | 26, 30.95 | 59, 23.69 | 0.19 |
| Raynaud’s phenomenon | 10, 11.90 | 28, 11.24 | 0.87 |
| Lymphatic system involvement | 6, 7.14 | 27, 10.84 | 0.33 |
| ESSDAI | 10.5 [6–16] | 7 [3–13] | 0.006 |

ESSDAI, European League Against Rheumatism Sjögren’s Syndrome Disease Activity Index; NC, normocomplementemia; HC, hypocomplementemia; pSS, primary Sjögren’s syndrome

**Sup-Table 2. Baseline characteristics of groups with and without hypocomplementemia at pSS diagnosis in terms of demographic and laboratory characteristics**

|  | **Total pSS** | **HC group**  **(n = 60)** | **NC group**  **(n = 186)** | ***P* value#** |
| --- | --- | --- | --- | --- |
|  | **(n=266)** |  |  |  |
| Prevalence of CD3 (+) in lymphocytes | 73.01[65.54-78.99] | 71.95[66.25-78.80] | 73.31[65.15-79.38] | 0.88 |
| Absolute number of CD3 (+) cells (/ul) | 1052.72 [750.97-1310.35] | 919.89 [669.03-1153.09] | 1100.67 [789.87-1331.01] | 0.02 |
| Prevalence of CD4 (+) in lymphocytes | 37.83[32.22-46.04] | 31.83[27.68-42.80] | 38.41[33.19-46.46] | 0.03 |
| Absolute number of CD4 (+) cells (/ul) | 553.52 [367.81-744.42] | 434.52 [308.82-632.49] | 579.54 [399.76-779.83] | 0.001 |
| Prevalence of CD8 (+) in lymphocytes | 28.17 [22.37-35.66] | 31.21 [23.51-39.25] | 27.37 [22.16-34.20] | 0.10 |
| Absolute number of CD8 (+) cells (/ul) | 376.70 [213.87-530.48] | 366.16 [255.02-545.59] | 378.55 [280.76-517.36] | 0.55 |
| Th/Tc | 1.37 [0.97-1.90] | 1.22 [0.72-1.62] | 1.45 [1.01-2.06] | 0.04 |
| Prevalence of CD19 (+) in lymphocytes | 11.96 [7.52-16.66] | 13.94 [7.33-17.12] | 11.62 [7.59-15.81] | 0.24 |
| Absolute number of CD19 (+) cells (/ul) | 166.38 [95.35-256.65] | 155.55 [84.96-240.14] | 167.29 [96.68-261.84] | 0.54 |
| Prevalence of CD16/CD56 (+) in lymphocytes | 13.05 [9.02-18.18] | 11.45 [7.14-16.08] | 13.36 [9.39-19.07] | 0.04 |
| Absolute number of CD16/CD56 (+) cells (/ul) | 178.63 [112.18-422.77] | 135.33 [78.99-221.78] | 190.94 [126.82-311.10] | 0.001 |
| HC, hypocomplementemia NC, normal complement;  # p value: HC group vs. NC group; | | | | |

Sup-Figure 3. CD3-CD16CD56+ NK cells in pSS. Shown are the CD3-CD16/CD56+NK cells representative two-dimensional scatter plots for an pSS patient.


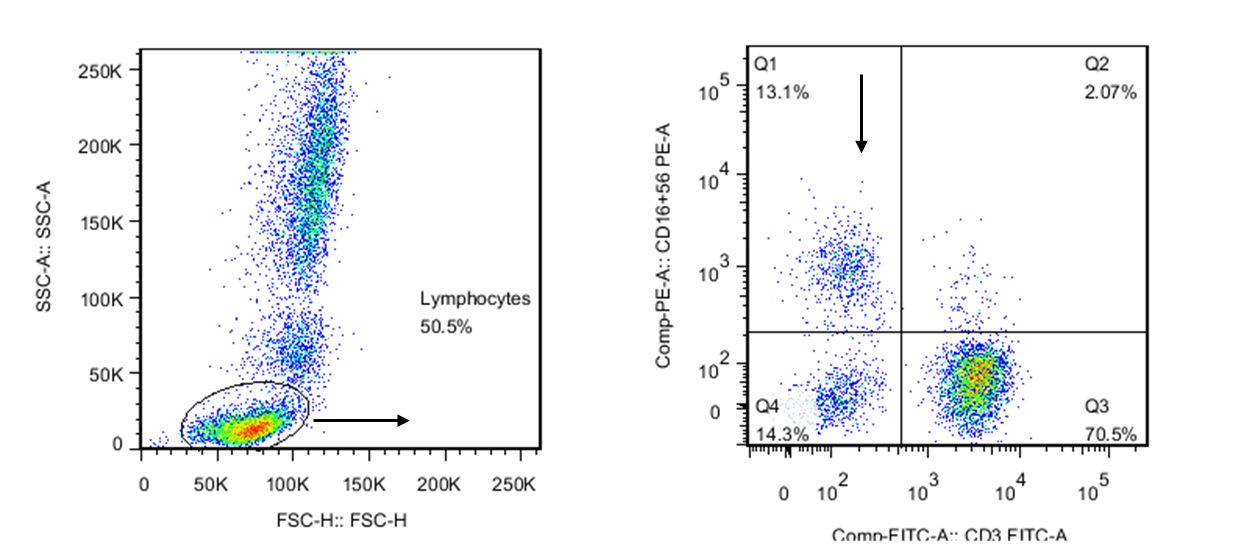

Supplement: Supplementary file 5 — (DOCX 251 kb) [file 10067_2022_6135_MOESM3_ESM.docx]
